# Cross-cultural adaptation and psychometric properties’ evaluation of the modern standard Arabic version of Cumberland Ankle Instability Tool (CAIT) in professional athletes

**DOI:** 10.1371/journal.pone.0217987

**Published:** 2019-06-11

**Authors:** Vasileios Korakakis, Mohsen Abassi, Argyro Kotsifak, Hassine Manai, Anas AbuEsba

**Affiliations:** Aspetar, Orthopaedic and Sports Medicine Hospital, Doha, Qatar; University of Texas at San Antonio, UNITED STATES

## Abstract

**Purpose:**

To cross-culturally adapt the Cumberland Ankle Instability Tool into modern standard Arabic and to assess its psychometric properties.

**Method:**

Cross-cultural adaptation followed a combination of guidelines and for psychometric evaluation a sample of 107 athletes as recruited. All recommended measurement properties by the Consensus-based Standards for the selection of health status Measurement Instruments were evaluated, including face, structural, convergent, and discriminant validity; reproducibility; distribution-based responsiveness, and interpretability. We also used a structured content analytic method to evaluate content validity.

**Results:**

The tool presented excellent internal consistency (α = 0.92) and reliability (ICC 0.75–0.98), and good convergent validity compared with Lower Extremity Functional Scale (ρ = 0.67). For reproducibility testing: Minimal detectable change ranged from 0.41 to 6.0 points; for responsiveness assessment: the effect sizes were large (Glass’*Δ* range 2.03–2.08, Cohen’s *d* range 2.22 to 2.53) and the Area under the Curve was 0.869. Its unidimensionality was proved by a 1-factor solution explaining 63.8% of the variance.

**Conclusion:**

The Arabic version of Cumberland Ankle Instability Tool presented acceptable psychometric properties comparable to the original version. The questionnaire is understood across most of the Arabic speaking world and can be used in research and clinical practice to assess patients suffering from chronic ankle instability.

## Introduction

Chronic ankle instability (CAI) is a common consequence of acute lateral ankle sprain (LAS). [1] Despite adequate initial treatment, more than 30% of patients with LAS will develop CAI which in turn leads to persisting complaints of “giving way”, recurrent LAS injuries, and pain. [2] Evidence indicates that balance, proprioception, reaction time and strength are impaired in CAI patients compared with healthy controls. However, the inclusion of heterogeneous participants in most of the studies limits the generalizability of the former to the entire “chronically unstable” population. [3] The International Ankle Consortium, based on the best available evidence, provided a position statement of selection criteria for patients with CAI to be used in future research. [4] The total score of the Cumberland Ankle Instability Tool (CAIT) was included among these criteria; a patient-rated outcome measure (PROM) aiming to determine the presence of functional ankle instability and to grade the severity of the instability. [5] The 9-item questionnaire inquires as to the degree of instability of performance of functional activities on Likert scales; the total score ranges from 0 to 30 with lower scores indicating more severe instability.

Despite that the number of available PROMs has increased dramatically over the past decades, most of these instruments are developed for English-speaking patients. PROMs in order to be used in different language and culture populations require a specific methodology with aim the adequate linguistic translation, but more importantly the cultural adaptation to maintain the content validity of the instrument across different cultures. [6] The CAIT has already been cross-culturally adapted in several languages. [7–13] CAIT utilizes lay terminology in simple English sentence format making it feasible to translate into modern standard Arabic (MSA) for a lay population. Regional Arabic dialects often create barriers to clear communication whereas MSA is widely used and understood in the Middle East and North Africa (MENA) region. Accordingly, a cross-culturally adapted questionnaire understood across most of the Arabic speaking world would yield the most practical and usable tool. Therefore, the main objectives of this study were: i) to cross-culturally adapt the CAIT for a wide spectrum of Arabic-speaking athletes with CAI, and ii) to evaluate its psychometric properties.

## Methods

The cross-cultural adaptation process adhered to published guidelines. [6, 14, 15] The CAIT’s assessment of psychometric properties followed quality criteria on the evaluation of health status questionnaires [16] and the Consensus-based Standards for the selection of health status Measurement Instruments (COSMIN) recommendations. [17]

This study conducted in our institutional rehabilitation department from May 2017 to March 2018. Ethics approval was obtained from the Institutional Review Board (Anti-Doping Lab Qatar—SCH-ADL-A-070) and all participants gave written informed consent.

### Translation and cross-cultural adaptation

The CAIT [5] was developed for English-speaking patients and translated into MSA. The process followed adapted steps from published recommendations [6, 14, 15] (Table 1) to ensure uniformity between source and target language.

**Table 1:** The steps of translation and cross-cultural adaptation of the CAIT questionnaire for Arabic-speaking patients.

Steps	Procedures
Step 1: *Initial Translation*	Two bilingual and bicultural translators, whose native language was Arabic, independently produced 2 translations and 2 written reports. One translator (informed) had medical background (physiotherapist) and was aware of the construct of the scale, while the other translator (naïve) had no clinical background (secretary), but was knowledgeable about the cultural and linguistic nuances of the Arabic language.
Step 2: *Reconciliation Committee*	A bilingual committee (4 physiotherapists and a sports medicine physician), a coordinator (researcher with several years of experience in scales development and validation), and the translators synthesized the 2 translations and through a consensus process harmonized and produced a common initial translation and a written report documenting the synthesis process. A bilingual member of the committee was recording changes and decisions.
Step 3: *Back Translation*	Two translators, whose native language was English and who were fluent in Arabic language, produced 2 independent back translations of the initial questionnaire. Both were uninformed of the concepts explored to avoid information bias and were blind to the original questionnaire. One of them had no medical background, while the other was a sports physician.
Step 4: *Back translation review committee and harmonization*	An independent committee (included bilingual clinicians knowledgeable about the content area) consisting of the translators, 3 bilingual clinicians, and two members of the research team convened, reached consensus, and developed the pre-final version of the CAIT-Arab for translation validation. During this process the committee assessed the original questionnaire [5] and each translation together with the corresponding written report. Special attention was given by the committee to ensure intertranslation validity and to achieve semantic, idiomatic, experiential, and conceptual equivalence between the source and target questionnaire. [6, 14, 15] Furthermore, the committee discussed “comparability of language” which refers to the formal similarity of words and sentences between the original and back-translated questionnaire, as well as “similarity of interpretability” which refers to the degree to which the versions produce the same response even if the wording differs. [6, 14, 15]
Step 5: *Validation of translation*	The validation of the CAIT-Arab regarding the success of the translation process was assessed in two ways: a) Formal evaluation of comparability of language and similarity of interpretability by using 7-point Likert scales ranging from 1 (extremely comparable/extremely similar) to 7 (not at all comparable/not at all similar). [14] Four bilingual individuals (2 men and 2 women, mean age 29 years and range 25 to 34 years) rated each original and back-translated item. Following this process each mean score >3 (comparability) and >2.5 (similarity) requires review for possible correction. b) Cognitive debriefing: the CAIT-Arab was tested for cognitive equivalence [15] by 7 native Arabic speaking patients representing the Gulf region population (Qatar, Jordan, Syria, Lebanon, Morocco, Tunisia, and Egypt) in order to capture the differences in Arabic dialects across the MENA region (mean age(range) 33.4(26–43) years). The same 7 individuals were also used for the item-content relevance analysis (see validity testing).
Step 6: *Review and finalization*	Based on comments from the former process the committee made all necessary modifications for improvement and checked the final version for spelling, diacritical, grammatical, or other errors.
Step 7: *Pretesting*	The pre-final version of the CAIT-Arab was administered to 15 Arabic-speaking athletes suffering from chronic ankle instability or lateral ankle sprain (men with age_(range)_ of 23.7_(18–32)_ years). Following the completion of the questionnaire, each individual was formally interviewed regarding the comprehension of items and the chosen response as part of the assessment of face and content validity. Upon completion of pre-testing a committee convened and the pre-final version without corrections was accepted as the final version of the CAIT-Arab questionnaire (S1 File).

### Sample size calculation and participants

Sample size calculation was based on the intraclass correlation coefficient (ICC) and the maximum width of the 95% confidence intervals (95%CI) from previous cross-cultural adaptation publications. [7–13] The formula used to calculate the sample size [18] was *n* = 16*p*(1−*p*)/*w*^2^, where *p* was the lowest expected ICC (0.826) and *w* was the maximum reported width (0.156) of the 95%CI. The minimum required sample size required was 95 individuals, but we recruited a bigger sample of professional athletes in order to ensure stability of the variance-covariance matrix in the dimensionality analysis [16] and to account for non-attendances at rehabilitation sessions or retest occasions, ensuring that reproducibility testing would be done in “stable” patients/individuals. [16] Four groups of participants that were not participants in the translation and cross-cultural adaptation process were included in the study: 60 patients with CAI (n = 30) or LAS (n = 30), as well as 47 asymptomatic “at risk” for ankle sprain basketball and football players (healthy group), and 60 patients with other lower limb injuries than ankle injuries or instability (i.e. muscle injuries, meniscal tears, knee sprains) to evaluate interpretability of the tool (Table 2). The participants were recruited through direct contact during their physiotherapy or training sessions. Participants had to be ≥18 years of age, speak Arabic as a first language, participating in at least 7 hours of physical activity per week, and willing to give informed consent. Reported standard inclusion criteria described in detail elsewhere [19] were used for CAI group (history of at least one significant ankle sprain, recurrent sprain, “feelings of instability and/or giving way”, CAIT≤24). Participants at LAS had a recent (≤4 days) lateral ankle sprain, while at risk-healthy individuals had no ankle injury the last year or instability in their lifetime. General exclusion criteria were no other lower limb injury, previous ankle fracture or surgery. The rest of the exclusion criteria in terms of injury or not, and type were related to respective group allocation.

**Table 2:** Descriptive characteristics of the participants in the study.

Groups	CAI (n = 30)	LAS (n = 30)	Healthy (n = 47)	Other injury (n = 60)
Age (years)	24.7±3.7	23.2±5.6	22.7±4.2	25.3±7.0
Height (cm)	182.8±5.8*	176.7±8.6	176.9±6.0*	176.0±8.6
Weight (kg)	73.7±8.7	73.2±8.4	70.6±7.5	70.4±12.6
CAIT-score^Ɨ^	14.5±5.7	12.4 ±7.8	29.2±1.8	27.7±3.0

Values are presented as mean ± SD

*Indicates statistically significant differences, p<0.05

^Ɨ^ Score at first administration of the CAIT

Note. The characteristics of the participants gathered by the investigators during the first day of the assessment and before the administration of CAIT.

Abbreviations: CAI, chronic ankle instability; LAS, lateral ankle sprain; Healthy, asymptomatic basketball players used as population at risk for ankle injury; Other injury, other lower limb injuries than ankle injuries or instability

### Procedures

The CAIT-Arab (S1 File) was administered to the athletes (n = 107) and completed twice within a range of 4 to 5 days (other than ankle injury group was excluded) in the presence of one of the investigators in order to standardize the procedure. On completion of the questionnaire if the investigator identified a missing item due to oversight the patient was asked to respond to the item. Based on previously published methodology [20, 21] we only included participants who self-rated their condition as unchanged at the second administration. Finally, the CAIT was administered a third time to both CAI and LAS groups by the same investigator, following a 6-week rehabilitation programme (S2 File).

### Validity testing

Face Validity [22–24] was assessed: a) in 3 reported steps of the translation and adaptation process, b) by the individuals that appraised the extent to which the instrument assessed their condition during pre-testing of the CAIT, and d) formally during the content analysis procedure (see content validity).

Content Validity [22, 24] was tested individually in each item of CAIT-Arab through a structured content analytic method. [25] The items were distributed to 7 judges (Arabic-speaking patients representing the MENA region population) during the fifth step of translation/adaptation process and another 6 judges (2 sport physicians, 2 physiotherapists, and 2 professional athletes; all holding higher degrees in relevant areas) after the pretesting process. The 13 judges matched each of the 20 items based on their content to a five-point Likert scale (1 = poor, 2 = fair, 3 = good, 4 = very good, or 5 = excellent match). Finally, the content validity was also assessed by the 15 patients participated in the pre-testing phase of CAIT-Arab.

A criterion scale does not exist for functional CAI, hence we evaluated convergent validity [22–24] by using Lower Extremity Functional Scale [26] (LEFS-MSAr) and expecting a moderate correlation (ρ = 0.50) with CAIT-Arab based on data and methodology of the scale development publication. [5] Patients with an ankle injury or instability (n = 60) during the initial assessment and before the administration of CAIT-Arab were asked to complete the LEFS-MSAr.

For criterion validity we used discriminant validity [22, 24] in order to evaluate whether CAIT could discriminate between individuals with and without functional ankle instability. History of ankle sprain was used as the discriminative measure [5] and we hypothesized that individuals without an ankle sprain would score close to the maximum possible score of the scale, while patients with CAI would score lower.

Structural validity [22, 24] of CAIT-Arab was tested by exploratory factor analysis (EFA) given that the unidimensionality of the tool has not been confirmed in previous studies. [5, 8, 11, 10] CAIT has been suggested to be a unidimensional scale, [5] however evaluation in other cross-cultural adaptations revealed two [10, 11] or 3-factor solutions. [8]

Construct validity was also evaluated by known groups validity using the contrasted-groups approach. [22, 24] We hypothesized that individuals at risk for an ankle injury will score significantly higher in CAIT compared to CAI, LAS and also other than ankle injuries groups.

### Reliability testing

Inter-item reliability [22, 24] was assessed by using Cronbach’s alpha coefficient (α) (coefficient α was planned to be calculated individually for all possible sub-scales). [24]

Test-retest reliability [16, 22–24] assessed to evaluate the temporal stability. The CAIT-Arab was administered twice (range 4–5 days) and the interval between administrations was long enough to ensure that participants do not recall their original responses, but short enough to ensure clinical stability of the condition.

### Utility evaluation

To review the acceptability and the ease of administration of the CAIT-Arab we recorded the percentage of unanswered questions and the time spent by the participants filling it out. [22, 23]

### Ceiling and floor effects

The CAIT-Arab would be considered to have ceiling and floor effects [16] if more than 15% of the patients scored the maximum and minimum possible score respectively. Relative to each item of the questionnaire ceiling and floor effects were considered to have occurred if at least 75% of the patients scored the maximum or minimum score to that item, respectively.

### Responsiveness

Responsiveness [16, 27, 28] reveals the ability of a questionnaire to detect clinically important changes over time. We used distribution-based methods for responsiveness assessment. The CAIT-Arab was administered to both CAI and LAS groups on two occasions 6 weeks apart. We hypothesized that clinically meaningful differences should be displayed in scores obtained by these patients as a result of rehabilitation.

### Interpretability

Interpretability [16, 24] is the degree to which one can assign qualitative meaning to a self-rated outcome measure’s quantitative scores or change in scores. We assessed interpretability of CAIT-Arab scores: a) by presenting normative scores of at risk for an ankle injury individuals, b) by comparing the scores of CAI and LAS patients between two time points following treatment of known efficacy. We hypothesize that CAIT-Arab scores will increase following rehabilitation and we expected at least a 3-point increase for a true difference according previously reported minimal clinically important change of the scale. [29]

### Statistical analyses

Statistical analyses were performed using SPSS v19.0. We used non-parametric tests in statistical analyses as CAIT is in the ordinal scale. The level of significance was set at p>0.05. Descriptive statistics were used to calculate the characteristics of the participants, the scores of CAIT-Arab and LEFS-MSAr questionnaires, the mean scores for each item for comparability of language and similarity of interpretability, acceptability, and the ceiling and floor effects. Missing values were listwise excluded. The other than ankle injury group of participants was used only for known groups and discriminant validity testing.

#### Validity testing

For item-content relevant analyses the judges’ ratings were evaluated based on the validation procedure of Aiken’s item-content validity coefficient (*V*). [30] The *V* statistic provides statistical significance of judges’ ratings about an item’s content-match with its construct and its values range from 0 to 1 (1 = perfect agreement). The values were then compared against a right-tailed binominal probability table provided by Aiken [30] (*V* scores >0.70 considered as having acceptable validity, p<0.01). Convergent validity was assessed with Spearman *rho* (*r*) between the scores obtained from CAIT-Arab and LEFS questionnaires. [26]

To explore the factorial validity of CAIT-Arab an EFA (principal axis factoring) with varimax rotation was used. Eigenvalues over 1 were chosen and extracted, and items loading more than 0.40 were regarded as loading on a specific factor. Items loading more than 0.40 on 2 factors were assigned to the factor with a higher correlation. [31] The group with other than ankle injuries was excluded from dimensionality assessment.

Discriminant validity (cutoff score of CAIT) was assessed with a receiver operating characteristic (ROC) curve and area under the curve (AUC). The most upper left point in the diagram represents the optimal cut-off change score, which most effectively discriminates between patients with ankle instability and those without. [32–34] Additionally, the AUC reflects the probability of correctly discriminating between unstable and stable ankles improved and non-improved patients. This area varies from 0.5 (the questionnaire does not discriminate more effectively than chance) to 1.0 (perfect discrimination). [33, 34]

Known groups differences were calculated using the Kruskal-Wallis test. For post hoc comparisons we used the Mann-Whitney *U*-test with appropriate Bonferroni correction for multiple testing, resulting from the formula *k(k– 1)/2*, where *k* is the number of groups (p_adj_ = 0.0083). The Wilcoxon test was used for within-group differences between administrations.

#### Reproducibility testing

The internal consistency of CAIT’s sub-scales was assessed by using Cronbach’s *α*. Values of ≥0.70 have been proposed as a measure of good internal consistency. [16] Reproducibility was evaluated by using both Spearman’s *rho* and 2-way random effects model Intraclass Correlation Coefficient, type agreement (ICC_2,1_), because systematic differences are considered to be part of the measurement error. [16, 35] As a measure of agreement the absolute measurement error was expressed as the standard error of measurement (SEM_Agreement_ = SD x √1-ICC), including the systematic differences in order to distinguish them from real changes, e.g., due to treatment or natural history. [16] In addition, the minimal detectable change (MDC_95_ = 1.96 x √2 x SEM) was calculated, which corresponds to the minimal within-person change in score that, with p<0.05, can be translated as a real change above measurement error. [16, 36] Bland-Altman methods were used to indicate absolute agreement for test–retest measurements including a scatter plot of differences between applications, with 95% limits of agreement (mean change in scores of repeated administrations). [37]

#### Responsiveness

There is still no consensus on the most suitable statistical analysis to assess responsiveness. [16, 28, 33, 38] The Wilcoxon test, using scores separated by 6 weeks was conducted to evaluate longitudinal validity (data also used for interpretability). Also, effect size (ES) by using both baseline and pooled standard deviation (SD) (for the purpose of interpretability) and standardised response mean (SRM) were calculated [28] and interpreted according to published recommendations (values of 0.20, 0.50, and 0.80 or greater represent small, moderate and large responsiveness, respectively). [39]

## Results

An overview of the measurement properties of CAIT from the present study and all studies assessing its psychometric properties [5, 7–13] are presented in Table 3.

**Table 3:** Summary of measurement properties of all adapted versions of CAIT questionnaire.

Measurement property	CAIT-Arab	CAIT-En [5]	CAIT-BrP[7]	CAIT-Sp-1[8]	CAIT-Sp-2[9]	CAIT-K[10)]	CAIT-P[11]	CAIT-J[12]	CAIT-D[13]
**Convergent validity**									
	LEFS	LEFS		SF-36_phys_	CAIT-En	SF-36_phys_	FAAM_ADL_	Karlsson	FAOS
				ρ = 0.241		ρ = 0.70	ρ = 0.41	score	
	ρ = 0.67	ρ = 0.50	N/R	p = 0.012	ICC = 0.91	p = 0.001		ρ = 0.604	*ρ* = 0.36–0.43
	p<0.001	p<0.01		SF-36_ment_	p<0.001	SF-36_ment_	FAAM_Sport_	p<0.001	p<0.0005
				ρ = -0.162		ρ = -0.06	ρ = 0.43		
				p = 0.094		p = 0.48			
**Discriminant validity**									
	Cutoff score≤23	Cutoff score≤27.5	N/R	N/R	N/R	N/R	N/R	Cutoff score≤23	Cutoff score≤11
	AUC = 0.87							AUC = 0.93	
	Sn = 1.0	Sn = 1.0						Sn = 0.705	Sn = 0.82
	Sp = 0.752	Sp = 0.752						Sp = 0.980	Sp = 0.91
**Factorial validity**									
Factor structure	1-factor solution	2-factor solution	N/R	3-factor solution	N/R	2-factor solution	2-factor solution	N/R	N/R
		Rasch analysis							
Variance %	63.8%			66.4%		74.5%	61.4%		
**Internal consistency**									
Cronbach’s α	0.92	0.83	0.86_R_ 0.88_L_	0.766	0.84_R_ 0.80_L_	0.89	0.81_R_ 0.79_L_	0.833	0.856
**Test-retest reliability**									
ICC	0.979	0.96	0.95	0.979	L:0.95	0.94	L:0.91	0.826	0.943
All (n = 107)	(95%CI: 0.969–0.986)	(95%CI:N/R)	(95%CI: 0.93–0.97)	(95%CI: 0.958–0.99)	(95%CI: 0.93–0.96)	(95%CI:N/R)	(95%CI: 0.80–0.94)	(95%CI: 0.73–0.89)	(95%CI: N/R)
ICC	0.873				R:0.95		R:0.95		
CAI (n = 30)	(95%CI: 0.751–0.937)				(95%CI: 0.94–0.97)		(95%CI: 0.91–0.97)		
ICC	0.968								
LAS (n = 30)	(95%CI: 0.932–0.985)								
**Test-retest reliability**									
Spearman’s rho	*ρ* = 0.986,	N/R	N/R	N/R	N/R	N/R	N/R	N/R	N/R
All (n = 107)	*p*<0.001								
Spearman’s rho	*ρ* = .868,								
CAI (n = 30)	*p*<0.001								
Spearman’s rho	*ρ* = .972,								
LAS (n = 30)	*p*<0.001								
**Agreement**									
SEM all (n = 107)	0.15	N/R	N/R	N/R	N/R	1.72	2.00_R_ 2.40_L_	N/R	0.82
SEM CAI (n = 30)	2.16								
SEM LAS (n = 30)	1.45								
**MDC**_**(95)**_									
MDC_in_ all (n = 107)	0.41	N/R	N/R	N/R	N/R	4.77*	5.60_R_ 6.50_L_	N/R	2.28
MDC_in_ CAI (n = 30)	6.00								
MDC_in_ LAS(n = 30)	4.02								
**Responsiveness**									
**CAI**		N/R	0.75	1.07_(Cohen *d*)_	0.70_(Cohen *d*)_	N/R	N/R	N/R	N/R
ES _baseline SD_	2.03_(Glass’ *Δ*)_								
ES _pooled SD_	2.22_(Cohen *d*)_								
SRM	1.73								
**LAS**									
ES _baseline SD_	2.08_(Glass’ *Δ*)_								
ES _pooled SD_	2.53_(Cohen *d*)_								
SRM	2.08								

* calculated by data given in publication

Abbreviations: CAIT, Cumberland ankle instability tool; LEFS, lower extremity functional scale; N/R, not reported; SF-36_phys_, short form 36 physical dimension; SF-36_ment_, short form 36 mental dimension; FAAM_ADL_, foot and ankle ability measure subscale of activities of daily living; FAAM_Sport_, foot and ankle ability measure subscale of sport activities; FAOS; Foot and Ankle Outcome Score; AUC, area under the curve; Sn, sensitivity; Sp, specificity; R, right limb; L, left limb; ICC, intraclass correlation coefficient; 95%CI, 95% confidence intervals; SEM, standard error of measurement; MDC_in_, minimal clinical change; CAI, chronic ankle instability; ES, effect size; SD, standard deviation; LAS, lateral ankle sprain; SRM, standardized response mean.

### Translation and cross-cultural adaptation

Minor linguistic discrepancies were easily resolved through the collaboration of content experts and native Arabic speakers of the committees during the consensus meetings. In item 5, the sentence “on the ball of my foot” could not be translated into Arabic and as a result the decision was made to partially abandon a literary description in favour of an image that clearly demonstrates the area referred to in the question.

### Validity testing

The face validity of CAIT-Arab was appraised as excellent from participants at pre-testing, patients with CAI and LAS, members of the expert committees, authors, and judges at item-content relevance testing. Construct validity was assessed through a structured content analytic method [25] and regarded as being well addressed by all 13 judges and 15 patients at pre-testing. All 9 items presented *V* values ranging from 0.83 to 0.98 (p<0.01).

A moderate significant correlation for convergent validity evaluation was found between LEFS-MSAr and CAIT-Arab (*rho* = 0.67, p<0.001) with a clear ceiling effect of the LEFS, as expected. [5]

The ROC curve showed that there was a distinct discrimination score (≤ 23 points) that can identify patients with an ankle sprain (AUC = 0.869, p<0.001). The maximum Youden’s index related to this cutoff score was 0.752, and had sensitivity of 100% a specificity of 75.2%, with a positive likelihood ratio of 4.0 and a negative likelihood ratio of 0.

**Fig 1:**
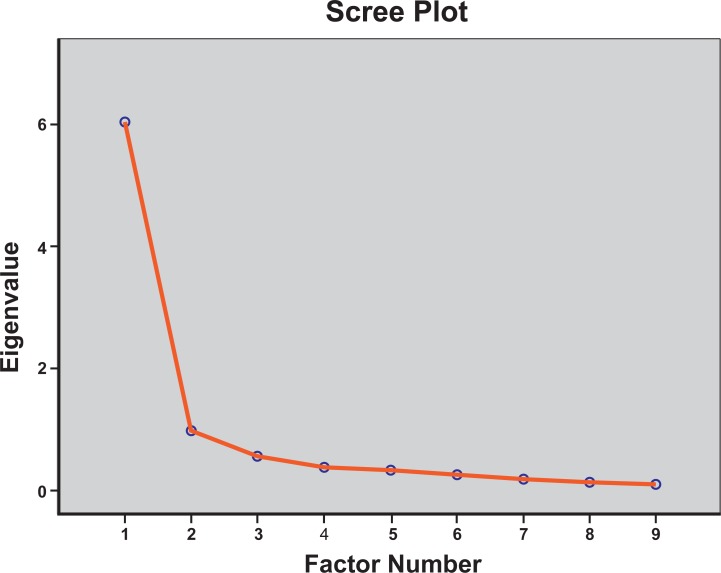
Exploratory factor analysis. Scree plot for CAIT-Arab dimensionality assessment depicting 1-factor solution.

Factorial validity testing extracted one-factor (Fig 1) with eigenvalues over Kaiser’s criterion of 1 explaining the 63.79% of total variance (Table 4) (KMO = 0.896, Bartlett’s sphericity test (*x*^*2*^_(36)_ = 811.613, p<0.001).

**Table 4:** Exploratory factor analysis with varimax rotation suggesting 1-factor solution for CAIT-Arab.

Item	Rotated factor loadings
7. My ankle feels UNSTABLE when (surface)	0.922
1. I have pain in my ankle	0.902
3. When I make SHARP turns, my ankle feels UNSTABLE	0.882
4. When going down the stairs, my ankle feels UNSTABLE	0.863
2. My ankle feels UNSTABLE when (sport, ADL)	0.834
5. My ankle feels UNSTABLE when standing on ONE leg	0.792
6. My ankle feels UNSTABLE when (hop, jump)	0.726
9. After a TYPICAL incident of my ankle rolling over, my ankle returns to “normal”	0.701
8. TYPICALLY, when I start to roll over (or twist) on my ankle, I can stop	0.462
Eigenvalues	6.05
Total Variance %	**63.79%**

Note: factorial analysis without the other than ankle injury group.

Abbreviations: CAIT, Cumberland ankle instability tool; ADL, activities of daily living

In terms of normative values for CAIT-Arab, Kruskal-Wallis tests revealed significant differences (p<0.001) for mean scores at first and second administration. No within group differences at Wilcoxon tests were found at both administrations (p>0.05). No significant differences were found in CAIT scores between CAI and LAS patients (p = 0.310). Both CAI and LAS group patients scored significantly lower (p<0.017) than both athletes at risk and patients with other lower limb injuries (Table 5).

**Table 5:** Total scores for the CAIT-Arab questionnaire at both administrations.

Group	N	Test*	Re-test*
CAI	30	14.5±5.7 (12.3–16.6); 16.0 (10)	14.2 ± 6.4 (11.8–16.6); 15.0 (12)
LAS	30	12.4±7.8 (9.5–15.3);12.0 (14)	13.1 ± 8.4 (9.9–16.2); 11.0 (14)
Healthy	47	29.2±1.8 (28.6–29.7); 30.0 (0)	29.2 ± 1.7 (28.7–29.7); 30.0 (0)
Other	60	27.7±3.0 (26.9–28.4); 29.0 (13)	N/A

*Data are presented as mean ± SD (95% CI) and as median (interquartile range) for interpretability reasons.

The Mann-Whitney test did not reveal significant differences between CAI and LAS groups at both assessments (U_test_ = 381.5, U_retest_ = 413.5.5, p = 0.310 and p = 0.589, respectively). CAI patients scored significantly lower at both assessments than healthy group (U_test_ = 0.0, U_retest_ = 1.0, both p<0.001) and other than ankle injuries group (U_test_ = 20.5, p<0.001). LAS patients scored significantly lower at both assessments than healthy group (U_test_ = 3.5, U_retest_ = 21.0, both p<0.001) and other than ankle injuries group (U_test_ = 38.5, p<0.001). Significant lower scores were found for patients with other than ankle injuries compared to healthy individuals (U_test_ = 969.0, p = 0.002).

Abbreviations: CAIT-Arab, Cumberland ankle instability tool Arabic version; N, sample size; CAI, chronic ankle instability group; LAS, lateral ankle sprain group, Healthy, asymptomatic at risk for ankle sprain basketball players; Other, other lower limb injuries; N/A, not applicable.

### Reliability testing

Reliability results are presented in Table 3. The Cronbach alpha for internal consistency was 0.92, and the Cronbach alpha if item deleted (for each item) varied from 0.91 to 0.92.

**Fig 2:**
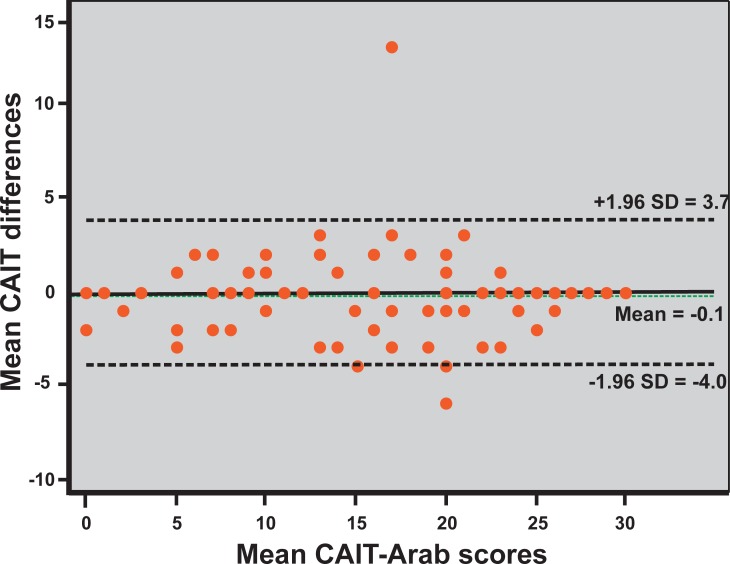
Bland-Altman plot. A Bland-Altman plot visualizing the agreement for test-retest with the limits marked as mean difference ±SD in a 30-point scale.

The Bland-Altman plot (Fig 2) showed no systematic bias; the mean differences were plotted around the zero line and within the limits of agreement (3.7 to -4.0) with a few outliers.

### Utility evaluation

The CAIT-Arab revealed a maximum response rate, which might be affected by the presence of one of the investigators. The completion of the questionnaire required 2 to 3 minutes revealing the ease of administration.

### Ceiling and floor effects

No ceiling effect was found for CAIT-Arab total score at first (0%) or second administration (0%), or a floor effect (0% respectively). Moreover, most of individual items of the scale were not scored at their maximum or minimum score by more than 75% of the patients at first administration (floor range 6.7–33.3%, ceiling range 3.3–36.7%) except item 9 that 80% of the participants scored the minimum score. Regarding or second assessment no individual items presented floor or ceiling effect by more than 75% of the patients (floor range 6.7–66.7%, ceiling range 3.3–36.7%).

### Responsiveness

The Wilcoxon test revealed statistically significant changes of the CAIT-Arab from first (Median = 16.0) to second (Median = 24.5) administration for this group (p<0.001) representing large effect sizes (Table 3).

Based on a change score equal or larger to the MDC_95_ (6 points) at the final administration of the LEFS-Arab, 80.0% of the patients were rated as improved, while 20% were found with no change.

### Interpretability

Normative scores of patients with ankle injury and of individuals at risk for an ankle injury are presented in Table 5. Statistically significant changes over time [pre-treatment (mean±SD = 14.5±5.7; median = 16.0) and post-treatment (mean±SD = 23.8±4.4; median = 24.5)] were found for CAIT-Arab (p<0.0001) with a large effect size (2.03).

## Discussion

The CAIT-Arab is a brief, valid and reliable outcome measure, available to be used across the MENA region in Arabic-speaking patients with CAI. As a psychometrically robust tool, it can be used to identify and assess the severity of CAI, as well as to evaluate outcomes for clinical and research purposes.

### Translation and cross-cultural adaptation

There are at least 12 major sets of guidelines available for questionnaires translation [15] and to our knowledge there is no consensus on a set of rigid procedures in the area of translation and cross-cultural adaptation. We implemented a rigorous adaptation process by following mixed methodology from published guidelines [6, 14, 15] and including content experts, bilingual and bicultural committee members, and native Arabic speakers. Also, we introduced a new step into the translation validation process using a formal evaluation of comparability of language and similarity of interpretability, [14] and testing for cognitive equivalence [15] involving native Arabic speaking patients representing the Arab population (Qatar, Jordan, Syria, Lebanon, Morocco, Tunisia, and Egypt). The process captured the differences in Arabic dialects and resulted in a widely comprehensible and practical tool for use across the MENA region. Finally, a problem with a phrase that could not be translated into Arabic was resolved by abandoning a literary description in favour of an image; a methodology previously applied in the cross-cultural research. [20, 21]

### Validity testing

As hypothesised, the CAIT-Arab demonstrated good translational and construct validity. Evaluation of content validity by a structured analytic method [25] added to the psychometric properties of CAIT, as to our knowledge this is reported first time since most of previous studies used floor and ceiling effects to examine this form of validity. [7, 9, 11, 13] The results also confirmed our hypothesis regarding the convergent validity of CAIT-Arab with the LEFS [26] scores presenting a moderate correlation as previously reported, probably because of a ceiling effect with the LEFS. [5] The ceiling effect of LEFS has been attributed to its insufficient sensitivity to identify problems related with CAI, as most of the tasks included in the scale are not sufficiently challenging for the lower leg. [5] Nonetheless, CAIT was only moderately correlated with SF-36, [8, 10] FAAM, [11] FAOS, [13] and Karlsson score [12] in previous studies, suggesting the administration of condition-specific outcome measures in clinical evaluation of CAI.

Guidelines suggest a confirmatory factor analysis for the assessment of PROMs’ structural validity in the presence of an existing theoretical model or because the factor structure has been determined previously. [16] However, given that previous analyses revealed both a 2-factor [10, 11] and a 3-factor solution [8] for CAIT, even though it has been suggested to be a unidimensional scale, [5] we decided re-explore its structure by using EFA. Internal consistency is an important measurement property for questionnaires that intend to measure a single underlying construct by using multiple items, [16] but it can be affected by the sample’s configuration. In the development publication of CAIT [5] the unidimensionality of the scale was not confirmed and this was attributed to the lack of homogeneity in the sample used in Rasch analysis. The EFA revealed that the CAIT-Arab was a unidimensional construct for functional CAI and this can be partially explained by the recruitment of a relatively homogeneous athletic population.

Hiller et al, [5] established a cut-off score of ≤27 as indicative of CAI that differs from the 23 points calculated in the present study. It has been rationally argued that with this cut-off value a patient could be classified as having an unstable ankle based only on a low score on the first question. [13] Additionally, in other studies evaluating CAIT’s psychometric properties this value was lower and ranged from 11 to 25 points. [12, 13, 40] A plausible explanation can be given by the fact that some studies [5, 12] used a history of ankle sprain alone to define group membership (CAI or not) when calculating the cutoff score instead of using self-reported ankle instability. [13, 40] Recently, the International Ankle Consortium [4] recommended that a cut-off score ≤24 should be used in CAI diagnostic criteria, a score consistent with the present report and confirmed by another relevant study. [40]

### Reliability testing

An excellent reliability was demonstrated for all participants (ICC = 0.97) in accordance with all previous studies (Table 3). The MDC reflects the smallest within-patient change in score that can be interpreted as a true change (i.e. because of treatment) beyond the measurement error. [16] The MDC of the CAIT-Arab score on the individual level was 6 points and was comparable to the MDCs reported (4.8 to 6.5) in studies used similar methodology. [10, 11] It must be noted that studies used subjectively experienced functional ankle instability to group and analyse participants’ data reported lower MDC values (2.28–3.08). [13, 29]

### Responsiveness

In the present adaptation we used only distribution-based methods to assess responsiveness and we acknowledge this as a limitation of the study. [16] Clinically meaningful score differences with large effect sizes, and a SRM of 1.73 were displayed, reflecting ability of CAIT-Arab to effectively distinguish changes over time. The ES reported here (2.03-Glass’ Δ and 2.22 Cohen’s d) is greater than that in other studies ranging from 0.70 to 1.07. [7–9] The 6-week interval between two evaluations used in the present study can probably explain the size of the difference with the other studies that treatment duration lasted 3–4 weeks. From a clinical perspective, treatment’s effect size is strongly affected by the interval between test-retest and must be interpreted with caution.

### Limitations

We acknowledge that the sample of patients with CAI was relatively small and of male gender. The extent to which our results can be generalized to female or non-professional athletes is unknown. Also, our methodology considered only classical test theory; given the inconsistency in available literature regarding the unidimensionality of the scale a rigorous Rasch analysis is much needed to re-examine in detail the internal structure of the CAIT. Finally, we acknowledge as a limitation of the study the use of distribution-based methods to assess responsiveness of the CAIT. These methods are considered measures to interpret changes in the condition, or to interpret the magnitude of the intervention, rather than measures of the quality of the instrument or the validity of the change score, therefore future studies should use anchor-based methods to evaluate responsiveness of the CAIT.

## Conclusions

The cross-cultural adaptation CAIT for Arabic-speaking patients with CAI was proven valid, reliable and responsive and can used for clinical and research purposes. In addition, to avoid barriers from regional Arabic dialects we cross-culturally adapted the CAIT into modern standard Arabic, resulting in a widely comprehensible and practical tool for use across the Middle East-North Africa region.

## Supporting information

S1 Data Anonymized data. (XLSX)

S1 File Modern standard Arabic version of Cumberland Ankle Instability Tool (CAIT). (PDF)

S2 File Acute first time and recurrent lateral ankle sprain rehabilitation guidelines. (DOCX)
