# Supplementary material for: Cross-cultural adaptation and psychometric properties’ evaluation of the modern standard Arabic version of Cumberland Ankle Instability Tool (CAIT) in professional athletes
Source: PLoS One. 2019 Jun 11;14(6):e0217987. doi: 10.1371/journal.pone.0217987 (PMC6559661; doi:10.1371/journal.pone.0217987)
Supplement: S1 File — (PDF) [file pone.0217987.s002.pdf]

inspired by aspire®

CUMBERLAND ANKLE INSTABILITY TOOL

[أداة كمبرلاند لعدم ثبات الكاحل]

The **CUMBERLAND ANKLE INSTABILITY TOOL** is not subjected to copyright and it's free to be used for clinical or research purposes. The ASPETAR version followed cross-cultural adaptation guidelines and was assessed for "comparability of language" and "similarity of interpretability".
The original publication to cite: Hiller CE, Refshauge KM, Bundy AC, Herbert RD, Kilbreath SL. The Cumberland Ankle Instability Tool: A Report of Validity and Reliability Testing. *Archives of Physical Medicine and Rehabilitation*. 2006;87:1235-1241.

For further information contact:
Mohsen Abassi (Mohsen.Abassi@aspetar.com) or
Vasileios Korakakis (Vasileios.Korakakis@aspetar.com)

أداة كمبرلاند لعدم ثبات الكاحل

يرجى وضع علامة على العبارة الوحيدة في كل سؤال لتصف كاحليك على النحو الأفضل.

	الأيسر	الأيمن	الدرجة
**1. لدى ألم في كاحلي**			
• أبداً			
• أثناء ممارسة الرياضة			
• الركض على أرضية غير مستوية			
• الركض على أرضية مستوية			
• المشي على أرضية غير مستوية			
• المشي على أرضية مستوية			
**2. أشعر بأن كاحلي غيرثابت**			
• أبدا			
• أحيانا أثناء ممارسة الرياضة (ليس في كل مرة)			
• بشكل متكرر أثناء ممارسة الرياضة (في كل مرة)			
• أحياناً أثناء النشاط اليومي			
• بشكل متكرر أثناء النشاط اليومي			
**3. عند قيامي بانعطافات حادة، أشعر بأن كاحلي غيرثابت**			
• أبدا			
• أحياناً أثناء الركض			
• غالباً أثناء الركض			
• أثناء المشي			

4. عند نزول الدرج، يبدو كاحلي غيرثابت

- أبدا
- إذا أسرعت
- أحياناً
- دائماً

5. يبدو كاحلي غيرثابت عند الوقوف على ساق واحدة

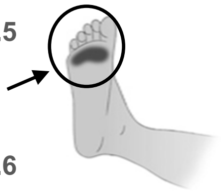

- أبداً
- على رأس القدم/ مقدمة القدم (ليس على أصابع القدم)
- وقدمي مسطحة تماماً

6. يبدو كاحلي غيرثابت عندما

- أبداً
- أقوم بالوثب من جانب إلى آخر
- أقوم بالوثب في مكاني
- أقفز

7. يبدو كاحلي غيرثابت عندما

- أبدا
- أركض على أرضية غير مستوية
- أهرول على أرضية غير مستوية
- أمشي على أرضية غير مستوية
- أمشي على أرضية مستوية

8. عادةً ، عندما يبدأ كاحلي في الإلتواء ، يمكنني إيقاف ذلك

- على الفور
- غالباً
- أحياناً
- أبداً
- لم يسبق أن إلتوى كاحلي

9. بعد إلتواء كاحلي، فإنه يصبح طبيعياً

- تقريباً على الفور
- أقل من يوم واحد
- من يوم إلى يومين
- أكثر من يومين
- لم يسبق أن إلتوى كاحلي
