# Supplementary material for: Cross-cultural adaptation and psychometric properties’ evaluation of the modern standard Arabic version of Cumberland Ankle Instability Tool (CAIT) in professional athletes
Source: PLoS One. 2019 Jun 11;14(6):e0217987. doi: 10.1371/journal.pone.0217987 (PMC6559661; doi:10.1371/journal.pone.0217987)
Supplement: S2 File — (DOCX) [file pone.0217987.s003.docx]

**Acute first time and recurrent Lateral Ankle Sprain Rehabilitation guidelines**

A sample illustration of the rehabilitation program can be found at the following link: https://youtu.be/MggDVaQoXAM

**Phase 1**

- Partial or full weight bearing as tolerated (with or without boot/crutches)
- Effusion control with soft tissue massage, ice, and light compression
- Multidirectional isometric contractions in neutral position of the joint
- Seated pain-free active range of motion by sliding on the floor (Plantar- and dorsi-flexion initially)
- Progress to seated pain-free active inversion/eversion
- Pain-free heel raises from different positions

**Phase 2**

- Normalize weight bearing status and gait, given that effusion is controlled
- ± taping for weight-bearing exercises and activities
- Static cycling without resistance
- Supported single-leg stance
- Full weight-bearing resisted (elastic bands) bilateral internal and external tibial rotations by sliding the forefeet and heels on the ground away and towards the starting position
- Full weight-bearing resisted (elastic bands), from mini-squat position, side-to-side walk, zig-zag forward and backward steps
- Bilateral heel raises (± elastic band resistance on rear-feet)
- Resisted (elastic bands) hip abduction in single-leg stance (bilateral)

**Phase 3**

- ± taping
- Static cycling with gradually increasing resistance
- Calf muscle stretching
- Lower limb muscle strengthening exercises
- Single-leg heel raises (straight and bent knee)
- Step ups (± external load)
- Decline squats (± external load)
- Lunges (± external load)
- Standing evertors pain-free eccentric loading
- Balance foam/disc progressive exercises (bilateral – progress to single-leg)
- Progressive functional strengthening on bridging position (on roller)
- Proprioception/balance training by using the “Balance Error Scoring System” – progress bilateral to single-leg to tandem stance (± surface and perturbation changes)
- Target oriented forward and backward step and foot placement
- Multidirectional hopping progressions (distance, vision available or obscured)
- Star excursion balance progressive drills

**Phase 4**

- Continue exercises from previous phases as needed
- Functional (± strength training) at end-range dorsi-flexed joint position
- Plyometric exercises
- Coordination drills (i.e. ladder)
- Running progression and sprinting (i.e. static, against the wall, on trampoline etc.)
- Changes of direction with speed and difficulty progression (i.e. figure of eight)

**Sports specific – phase 5**

- No deficit in range of motion, strength, proprioception as compared to the uninjured side (high patient confidence)
